# Underdiagnosis and underreporting of vertebral fractures on chest radiographs in men aged over 50 years or postmenopausal women with and without type 2 diabetes mellitus: a retrospective cohort study

**DOI:** 10.1186/s12880-022-00811-8

**Published:** 2022-05-01

**Authors:** Ding Na, Ma Cong, Wen Zhang-Xin, Chen Rong, Wang Qin-Yi, Ou Yang-Na, Sheng Zhi-Feng

**Affiliations:** 1 Health Management Center, National Clinical Research Center for Metabolic Diseases, Hunan Provincial Key Laboratory of Metabolic Bone Diseases, Department of Metabolism and Endocrinology, The Second Xiangya Hospital of Central South University, 139 Middle Renmin Road, Changsha, 410011 Hunan China; 2 Department of Radiology, The Second Xiangya Hospital of Central South University, Changsha, 410011 Hunan China; 3 Hospital Infection Control Center, The Second Xiangya Hospital of Central South University, 139 Middle Renmin Road, Changsha, 410011 Hunan China

**Keywords:** Vertebral fracture, T2DM, Prevalence, Underdiagnosis, Chest radiographs

## Abstract

**Background:**

Osteoporotic vertebral fractures are often clinically silent and unrecognized. The present study aimed to determine whether routine chest radiographs could be a potential screening tool for identifying missed vertebral fractures in men aged over 50 years or postmenopausal women, especially those with type 2 diabetes mellitus (T2DM). In this study, we aimed to determine the prevalence of undetected vertebral fractures in elderly Chinese patients with and without T2DM.

**Methods:**

Clinical data and chest radiographs of 567 individuals with T2DM (T2DM group) and 583 without diabetes (nondiabetic group) at a tertiary hospital in central south China were extracted from the records. Vertebral fractures were specifically looked for on chest radiographs and classified using the Genant semi-quantitative scale. Prevalence was compared between the two groups.

**Results:**

Mean age and sex composition were comparable between the two groups. Mean weight and body mass index were significantly lower in the T2DM group. In both groups, fractures mostly involved the T11–12 and L1 vertebrae. Moderate/severe fractures were identified in 33.3% individuals in the T2DM group (31.4% men and 36.0% women) versus 23.2% individuals (20.9% men and 25.5% women) in the nondiabetic group.

**Conclusions:**

Routine chest radiographs could be a useful screening tool for identifying asymptomatic vertebral fractures.

*Trial registration* The study was designed as an observational retrospective study, therefore a trial registration was not necessary.

## Introduction

Vertebral fractures are the most common type of osteoporotic fracture [1]. Occurring mostly in postmenopausal women and the elderly, these fractures are a cause of serious disability and even mortality [2–4]. An existing vertebral fracture increases the risk of a subsequent hip fracture by as much as 3.4-fold and that of new vertebral fractures by 12.6-fold [5, 6], but currently available treatments can reduce the risk by 40–50% [7, 8]. Therefore, early detection and subsequent treatment of vertebral fractures have a potentially beneficial impact on health care. Early detection and treatment are therefore important. Unfortunately, because many vertebral fractures are asymptomatic or only mildly painful, the diagnosis is often missed [9, 10]. Another reason is that confirmation of a suspected vertebral fracture requires radiography of the thoracolumbar spine [11]. However, clinical guidelines recommend thoracolumbar radiography only for patients with trauma, osteoporosis, malignancy, or acute back pain. A substantial proportion of vertebral fractures therefore go undetected [12].

The risk of osteoporotic vertebral fractures is reported to be high in patients with type 2 diabetes mellitus (T2DM) [13–15], especially in Asian populations [16, 17]. Vertebral fracture have independent adverse impact on daily activities and quality of life and is also independently associated with all-cause mortality in patients with T2DM [18, 19]. One recent study even suggested that vertebral fracture should be considered a complication of diabetes [20].

Previous studies have shown that routine chest radiographs obtained in different clinical settings can be a cost-effective way for identifying undiagnosed vertebral fractures [21, 22]. However, radiologists and clinicians often overlook old vertebral fractures, particularly if they are unrelated to the reason for the current presentation [21, 23]. This retrospective study aimed to determine the prevalence of undetected vertebral fractures in elderly Chinese patients with and without T2DM. To our knowledge, this is the first study of its kind in a Chinese population.

## Methods

### Study design and participants

This observational retrospective study was conducted at the second Xiangya hospital, a tertiary care hospital in China. Individuals with T2DM for whom front and lateral chest radiographs had been obtained from January 2018 to January 2021 were identified from the hospital records and included in the study. Individuals without T2DM were generally healthy individuals who had undergone a routine health checkup at our hospital at the same time. We excluded men younger than 50 years and premenopausal women. We also excluded patients who had a history of and/or whose radiographs indicated vertebral fractures due to high-energy trauma (n = 2), post-traumatic deformity (n = 6), metastatic tumors (n = 20), tuberculosis (n = 2), Scheuermann’s disease (n = 1), congenital spine deformity (n = 3), deformity due to degenerative scoliosis (n = 17), or whose lateral thoracic spine image was unclear (n = 29). If multiple medical records were available for the same individual, only the latest record was selected. A total of 1150 patients who met the eligibility criteria were selected for the study: 567 with diabetes (T2DM group) and 583 without diabetes (nondiabetic group).

### Data collection

#### Medical history and anthropometric information

The admission records and discharge summaries were retrieved, and data were collected on patient age and sex, height, weight, duration of diabetes, medication history, radiography findings, and discharge diagnoses. Body mass index (BMI) was calculated as weight in kilograms divided by height in meters squared.

#### Biochemical parameters

Fasting plasma glucose (FPG), glycated hemoglobin (HbA1c), serum 25(OH)D level, and serum creatinine level were recorded. FPG was measured using an automatic biochemistry analyzer (Hitachi 7360; Hitachi Ltd., Tokyo, Japan). HbA1c was measured using a hemoglobin testing system (Variant II, Bio-Rad, Hercules, CA). Serum 25(OH)D level was measured by quantitative sandwich enzyme-linked immunosorbent assay (Immunodiagnostic Systems Limited, Boldon, UK). Serum creatinine level was measured using an enzymatic method (Kanto Chemical, Tokyo, Japan), and the estimated glomerular filtration rate (eGFR) was calculated using the Chronic Kidney Disease Epidemiology Collaboration (CKD-EPI) 2009 equation.

### Identification of vertebral fracture

Vertebral fractures between the T4 thoracic spine and L2 lumbar spine were identified and classified using the Genant semiquantitative scale as grade 2/moderate fracture (vertebral height loss of 25–40%) or grade 3/severe fracture (vertebral height loss > 40%). A previous study reported the results obtained with these methods for an evaluation of 100 chest radiographs for prevalent vertebral fractures, and included assessments of interrater reliabilities across 3 different study radiologists (simple agreement, 87–89%; κ = 0.56–0.58) and between a reference standard radiologist and quantitative digital vertebral morphometry (simple agreement, 89%; κ = 0.67) [23]. The fractures were diagnosed by two radiologists (each with 9 and 12 years of experience in chest imaging) with the accreditation Certified Clinical Densitometrist conferred by the International Society for Clinical Densitometry (ISCD). Chest radiographs (FLUOROSPOT Compact FD, Siemens Healthineers; Carestream Health, Rochester, NY, USA) of all patients were bookmarked in the hospital’s digital archiving system according to their registered numbers. The registered numbers is a unique number assigned to the patient when they enter the hospital. We provided radiologists with registered numbers from lowest to highest. Two radiologists read both the front and lateral radiographs. This allowed study reviewers to independently view the radiographs while blinding them to official radiologist reports as well as other clinical data. If the diagnosis was inconsistent, the same two radiologists reassessed the image. If there was still no agreement, the case was classified as a non-fracture [21]. If the case was classified as grade 2 by one radiologists and grade 3 by another radiologist, we classified it as grade 2.

### Statistical analysis

Continuous variables were summarized as means ± standard deviation and compared between groups using the independent-samples *t*-test or the Mann–Whitney *U* test. Categorical variables were summarized as percentages and compared between groups using the chi-square test or independent-samples nonparametric test. Logistic regression analysis was performed to identify the risk factors for vertebral fractures. SPSS 23.0 (IBM Corp, Armonk, NY, USA) was used for statistical analysis. *P* < 0.05 was considered statistically significant.

## Results

The study controlled for the sex distribution and the mean age between the T2DM and nondiabetic groups to minimize the effects of sex and age. Table 1 shows the comparison of parameters between the T2DM group and the nondiabetic group by sex. Sex composition and mean age were comparable between the two groups (T2DM group: 328 men with mean age 60.8 ± 8.4 years and 239 women with mean age 63.1 ± 7.7 years vs. nondiabetic group: 340 men with mean age 61.1 ± 8.1 years and 243 women with mean age 63.2 ± 7.5 years). Mean weight and BMI were significantly lower in the T2DM group than in the nondiabetic group among both men and women. As is to be expected, HbA1c and FPG were higher in the T2DM group. The serum 25(OH)D level was significantly lower in T2DM men than in nondiabetic men (44.0 ± 17.1 ng/mL vs. 47.6 ± 17.8 ng/mL, *P* = 0.001); the 25(OH)D level was lower in T2DM women than in nondiabetic women, but the difference was not statistically significant. The prevalence of vertebral fractures was significantly higher in the T2DM group than in the nondiabetic group: 103 (31.4%) men and 86 (36.0%) women in the T2DM group versus 71 (20.9%) men and 62 (25.5%) women in the nondiabetic group. Only in two cases in each group were the fractures mentioned in the initial radiological report. However, the discharge records of these four patients did not mention vertebral fracture.

**Table 1:** Baseline characteristics of the participants

	Men		Women	
	Type 2 diabetes mellitus (n = 328)	Non-diabetic (n = 340)	Type 2 diabetes mellitus (n = 239)	Non-diabetic (n = 243)
Age (years)	60.8 ± 8.4	61.1 ± 8.1	63.1 ± 7.7	63.2 ± 7.5
Height (cm)	166 ± 6	166 ± 6	153 ± 5	154 ± 6
Weight (kg)	63.2 ± 8.3	68.2 ± 9.8*	52.4 ± 7.9	57.0 ± 8.5*
BMI (kg/m^2^)	23.0 ± 2.4	24.6 ± 2.9*	22.3 ± 3.0	24.0 ± 3.0*
eGFR (mL/min/1.73 m^2^)	87.2 ± 33.0	91.9 ± 19.2	88.3 ± 32.9	101 ± 24.8*
HbA1c (%)	8.81 ± 2.31	6.08 ± 1.07*	8.86 ± 2.39	6.02 ± 0.84*
FPG (mmol/L)	7.20 ± 2.82	5.77 ± 2.77*	7.48 ± 2.94	5.44 ± 1.39*
25(OH)D (ng/mL)	44.0 ± 17.1	47.6 ± 17.8*	40.1 ± 17.0	42.0 ± 14.6
Sulfonylurea n (%)	72 (22.0)	–	62 (25.9)	–
Metformin n (%)	115 (35.1)	–	88 (36.8)	–
Insulin n (%)	163 (49.7)	–	130 (54.4)	–
VF in the radiography report review n (%)	103 (31.4)	71 (20.9)*	86 (36.0)	62 (25.5)*
VF in the radiography report (n)	1	0	1	2
VF in the discharge report (n)	0	0	0	0

*BMI* body mass index, *eGFR* estimate glomerular filtration rate, *HbA1c* hemoglobin A1c, *FPG* fasting plasma glucose, *25 (OH) D* 25-hydroxyvitamin D, *VF* vertebral fracture

*Comparison with Type 2 diabetes mellitus, *P* < 0.05

**Fig. 1:**
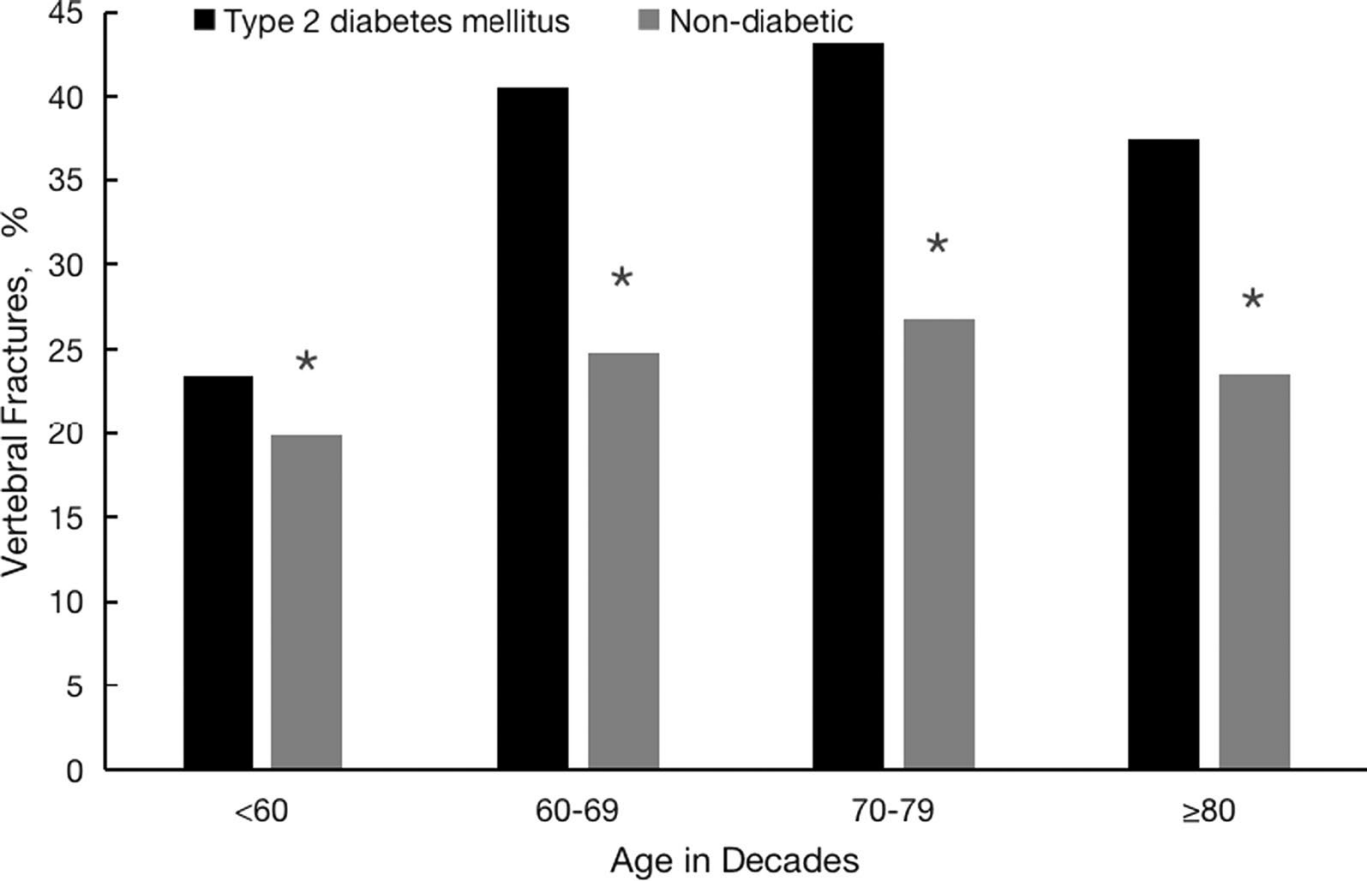
Prevalence of vertebral fractures, stratified according to age and with or without diabetic. *Comparison with type 2 diabetes mellitus, *P* < 0.05

Figure 1 displays the prevalence of vertebral fractures in the two groups, stratified by age. The prevalence tended to increase with age in both groups, though there was a slight decrease the oldest age-group of ≥ 80 years. Among both T2DM and nondiabetic patients, the prevalence of vertebral fractures were significantly higher in the 60–69 years age-group and 70–79 years age-group than in the 50–59 years age-group (*P* < 0.05), While the prevalence was higher in those aged ≥ 80 years than in those aged 50–59 years, the difference was not statistically significant. In addition, the prevalence of vertebral fractures was significantly higher in T2DM patients than in nondiabetic patients in all age-groups.

**Fig. 2:**
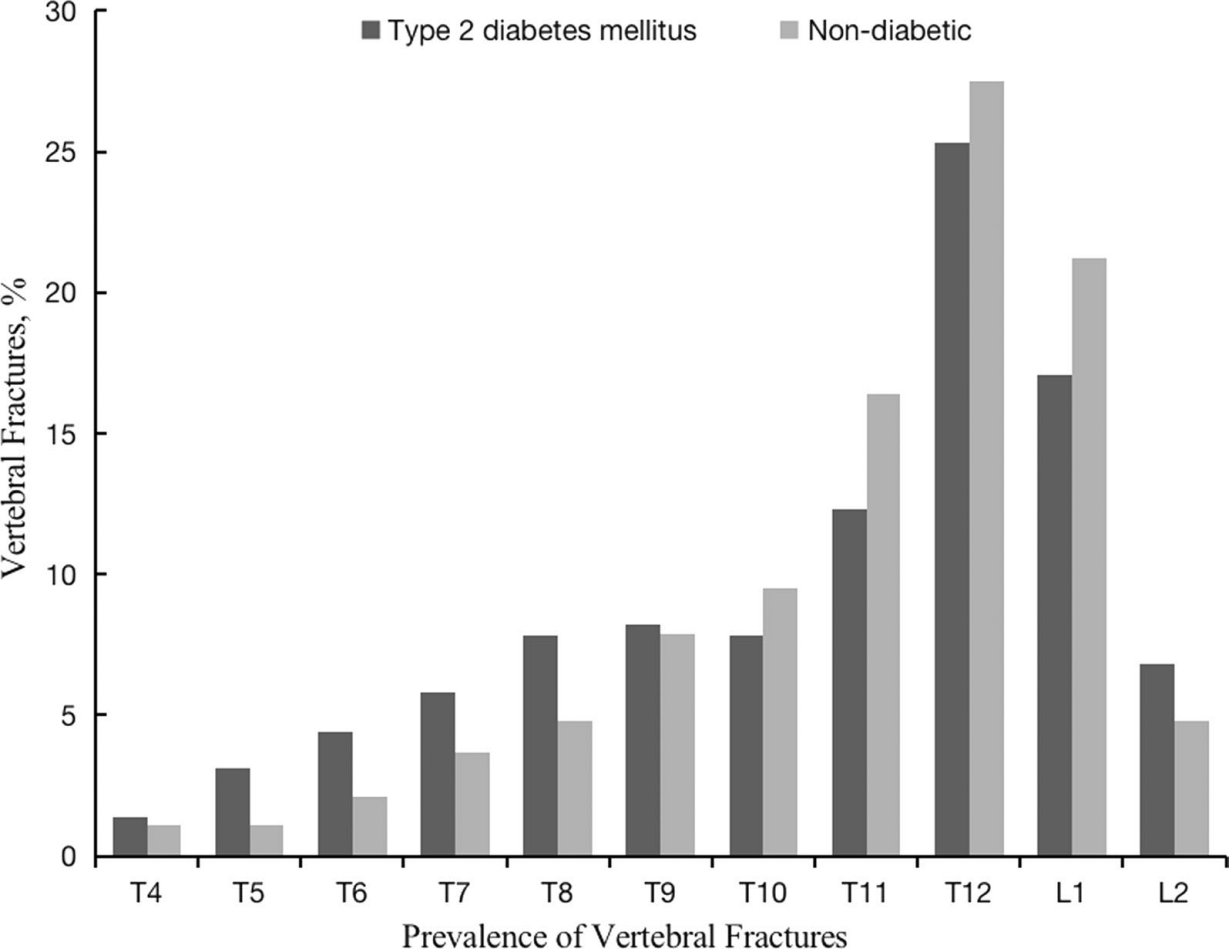
Prevalence of vertebral fractures at different position

Both moderate and severe vertebral fractures were more common in the T2DM group than in the nondiabetic group (Table 2). Among the 189 (33.3%) T2DM patients with moderate or severe fractures, 121 had only one fracture and 68 had two or more fractures. Among the 133 (22.8%) nondiabetic individuals with vertebral fractures, 101 had only one fracture and 32 had two fractures. In both groups, the fractures mostly involved T11–12 and L1. Overall, T12 was the vertebra most commonly involved (Fig. 2).

**Table 2:** Characteristics of vertebral fractures identified on chest radiographs

	Type 2 diabetes mellitus			Non-diabetic		
	Number of patients	Moderate vertebral fracture	Severe vertebral fracture	Number of patients	Moderate vertebral fracture	Severe vertebral fracture
Single fracture	119	105	14	101	99	2
Two or more fractures	68	58	10	32	30	2
Total	187	163	24	133	129	4

Logistic regression analysis (Table 3) showed age to be significantly associated with vertebral fracture in both sexes in the T2DM group (men: OR = 1.041, 95% CI: 1.012–1.069, *P* = 0.005; women: OR = 1.037, 95% CI 1.002–1.074, *P* = 0.041). In the nondiabetic group, age was significantly associated with vertebral fractures in men (OR = 1.028, 95% CI: 1.008–1.050, *P* = 0.007) but not in women (OR = 1.023, 95% CI: 0.998–1.050, *P* = 0.077). However, in this group, a high HbA1c was significantly associated with higher odds for the presence of vertebral fracture (OR = 1.099, 95% CI: 1.012–1.194, *P* = 0.025). BMI and serum 25(OH)D level were not associated with vertebral fracture. In men in the nondiabetic group, higher eGFR levels were significantly associated with lower odds for the presence of vertebral fracture (OR = 0.989, 95% CI: 0.982–0.997, *P* = 0.007).

**Table 3:** Association of various parameters with the presence of vertebral fractures

	Type 2 diabetes mellitus			Non-diabetic		
	OR	95% CI	*P*	OR	95% CI	*P*
Men						
Age (years)	1.041	1.012–1.069	0.005	1.028	1.008–1.050	0.007
BMI (kg/m^2^)	1.042	0.945–1.150	0.409	0.990	0.930–1.054	0.755
eGFR (mL/min/1.73 m^2^)	0.992	0.983–1.001	0.070	0.989	0.982–0.997	0.007
HbA1c (%)	0.997	0.900–1.105	0.085	1.065	0.989–1.148	0.097
FPG (mmol/L)	0.922	0.841–1.011	0.083	0.983	0.922–1.049	0.610
25(OH)D (ng/mL)	0.985	0.971–0.099	0.051	0.996	0.986–1.006	0.470
Women						
Age (years)	1.037	1.002–1.074	0.041	1.023	0.998–1.050	0.077
BMI (kg/m^2^)	1.006	0.920–1.100	0.899	0.961	0.902–1.024	0.225
eGFR (mL/min/1.73 m^2^)	0.999	0.989–1.009	0.876	0.999	0.992–1.007	0.863
HbA1c (%)	1.092	0.977–1.220	0.121	1.099	1.012–1.194	0.025
FPG (mmol/L)	0.969	0.880–1.067	0.520	0.993	0.916–1.076	0.856
25(OH)D (ng/mL)	0.995	0.980–1.011	0.571	0.993	0.981–1.006	0.282

*OR* odds ratio, *CI* confidence interval, *BMI* body mass index, *eGFR* estimate glomerular filtration rate, *HbA1c* hemoglobin A1c, *FPG* fasting plasma glucose, *25 (OH) D* 25-hydroxyvitamin D, *VF* vertebral fracture

*Comparison with Type 2 diabetes mellitus, *P* < 0.05

## Discussion

The fact that many vertebral fractures go undiagnosed [9, 10], and the accumulating evidence that patients with diabetes mellitus have increased risk of osteoporotic fractures [16] emphasizes the need for better methods to identify (and treat) vertebral fractures. The standard chest radiograph, ordered for various clinical indications, is a potential tool for detection of undiagnosed osteoporotic vertebral fractures. Although it is by no means the study of choice for examining the spine, a chest radiograph is adequate for identifying most osteoporotic fractures as these fractures mostly involve the mid-thoracic spine (T7–T8) and the thoracolumbar junction (T12–L1) [24], regions that are adequately visualized on routine chest radiographs. An additional advantage is that these radiographs have already been ordered for other purposes, so there is no added financial burden on the patient.

In this study, the prevalence of 22.8% that we found among patients without diabetes is very close to the 22% reported in a previous study that included 100 elderly patients presenting to a tertiary care emergency department and receiving chest radiography [23]. Meanwhile, the prevalence of 33.3% among patients with T2DM in our study, is slightly lower than the 38.7% found in a previous study from Japan that included 808 hospitalized T2DM patients aged ≥ 50 years [20]. The lower prevalence in our cohort may have been because we only considered moderate or severe vertebral fractures while the Japanese study considered mild to severe vertebral fractures. Furthermore, our patient population was younger, with mean age of 61.8 ± 8.2 years versus the mean age of 66.7 ± 9.1 years in the Japanese study [20]. The prevalence of vertebral fracture increases markedly with age [25], as was also confirmed in our study. Age was also significantly associated with vertebral fractures in logistic regression analysis. Finally, the prevalence of moderate/severe vertebral fractures was significantly higher in patients with T2DM than in those without diabetes. The pathophysiological mechanisms underlying bone fragility in T2DM are complex, and include hyperglycemia, oxidative stress, and the accumulation of advanced glycation endproducts that compromise collagen properties, increase marrow adiposity, release inflammatory factors and adipokines from visceral fat, and potentially alter osteocyte function. Additional factors include treatment-induced hypoglycemia, certain antidiabetic medications that exert direct effects on bone and mineral metabolism (such as thiazolidinediones), as well as an increased propensity for falls, all of which contribute to the increased fracture risk in patients with T2DM [14, 20].

In this study, the prevalence of vertebral fractures increased with age at most ages, but this trend disappeared after age 80. These results are similar to those of other studies [26, 27]. A possible explanation for our findings of low incident rates in oldest age-group of ≥ 80 years is that incidental vertebral fractures in this population were mainly contributed to osteoporosis rather than by strenuous physical activity and work. Previous studies suggested that the pathogenesis of fracture may be different in different age groups [27]. Osteoporosis may be the main cause of vertebral fractures in the elderly, while injury may be the main cause of vertebral fractures in the young. The population in the study was relatively young, with very few people over the age of 80. Moreover, many of them were still engaged in strenuous physical activity or work when they were enrolled in the study. Another possible explanation could be attributed to the more frequently in poorer health and higher mortality rate among those over 80 years of age. Previous studies have shown that bone mineral density increases again after about 78 years of age [28], and almost all types of fractures have an decreased incidence with high BMD [29].

In the present study, 98.9% of the vertebral fractures were not mentioned in the original radiology reports, and none were mentioned in discharge records. This under reporting is not unique to our institution. A study from the US found moderate-to-severe vertebral fractures in 132 (14%) of routine chest radiographs of 934 postmenopausal women aged over 60 years; only 17 (13%) of the discharge summaries documented these fractures [22]. One study that evaluated chest radiographs of 100 randomly selected patients aged ≥ 60 years presenting to the emergency department of a tertiary care hospital found 22 (22%) patients had moderate-to-severe vertebral fractures; however, only 12 (55%) of these fractures were mentioned in the radiology reports [23]. Another study of chest radiographs of 500 patients aged > 60 years attending an emergency department found that 72 (14%) patients had vertebral fractures, but only 43 (60%) of these fractures were reported by the radiologist, and only 18 (25%) patients with fractures received a diagnosis of or treatment for osteoporosis [21]. A retrospective study of 10,291 postmenopausal women aged over 60 years showed that while 142 (1.4%) of the original radiology reports mentioned a vertebral fracture, only 23 (16%) discharge summaries documented the fracture [30]. Another recent retrospective study of 3216 hospitalized female patients aged ≥ 50 years found previously undiagnosed vertebral fractures in routine chest radiographs of 67% of the patients [31]. Thus, it is obvious that under diagnosis of vertebral fracture is a common problem worldwide. A possible explanation is that radiologists tend to focus on the immediate serious illness and ignore the incidentally discovered vertebral fractures; alternatively, the under diagnosis may be because the radiologists lack sufficient experience and training for vertebral fracture recognition. Clinicians, on their part, may miss the fracture unless their attention is drawn to it by the radiologist. Thus, both radiologists and clinicians need to realize the importance of timely recognition of vertebral fractures on chest radiographs.

T2DM patients showed a higher prevalence of vertebral fractures than the control participants, and vertebral fractures occurred primarily in the thoracic spine T11–12 and lumbar spine L1 in both T2DM and control groups. These results are consistent with the findings of other studies, where fractured vertebrae were identified with greater frequency in the thoracic spine T11–12 and lumbar spine L1 in both elderly [31] and elderly patients with T2DM [20]. Vertebral fractures occurred primarily in the thoracolumbar junction. Thus, radiologists should focus on the thoracolumbar junction when diagnosing vertebral fractures.

This study has certain limitations. First, we considered only moderate-to-severe vertebral fractures, so it is possible that we underestimated the prevalence of vertebral fractures. However, we decided to exclude lower-grade fractures because mild vertebral deformities such as physiological wedging in the mid-thoracic region and short vertebral height may occur due to old age and degenerative changes [31]. Further, higher-grade fractures are associated with increased risk of future fracture, are better suited to semiquantitative techniques, and have better intraobserver and interobserver reliability and specificity [21, 23]. Second, the T2DM patients in this study were those seeking treatment for diabetes at a tertiary hospital; they probably had relatively severe diabetes and so might not be representative of all Chinese patients with diabetes. Third, most of the patients were on other treatments (including hypoglycemic drugs); we cannot exclude the possibility that the treatments affected the occurrence of vertebral fracture [20]. Fourth, all study participants were Chinese; the findings may not be applicable to other populations [32]. Fifth, other important risk factors for fractures (e.g., family history, smoking, and alcohol use) were not considered in this analysis [20]. However, it was almost impossible to collect data for all risk factors, and the implementation of a clinical assessment index consisting of complicated factors was also deemed to be impractical. Finally, this study was obviously limited by its retrospective design; our findings must be confirmed in longitudinal studies.

Despite these limitations, this study has several strengths. This is the first survey of vertebral fractures on routine chest radiographs in elderly patients with or without T2DM in the Chinese population, and we found that routine chest radiographs can effectively detect vertebral fractures. In addition, we also found a large gap between the recognition and diagnosis of vertebral fractures in clinical practice. Lastly, the findings highlight the opportunity for healthcare systems to develop interventions to improve the quality of care by undertaking strategies for elderly patients with or without T2DM who have had chest radiographs taken and who are found to have incidental vertebral fractures.

In conclusion, routine chest radiographs could be a useful tool for detecting vertebral fractures in Chinese patients. These fractures mostly involve the T11–12 and L1 vertebrae and tend to be more common in individuals with T2DM. However, a large proportion of these fractures go unrecognized. Thus, radiologists should be alert to the possibility of unsuspected thoracolumbar vertebral fractures when interpreting routine chest radiographs.

## Data Availability

All data generated or analyzed during this study are included in this published article.
